# Biofilm Formation by Chromoblastomycosis Fungi *Fonsecaea pedrosoi* and *Phialophora verrucosa*: Involvement with Antifungal Resistance

**DOI:** 10.3390/jof8090963

**Published:** 2022-09-15

**Authors:** Ingrid S. Sousa, Thaís P. Mello, Elaine P. Pereira, Marcela Q. Granato, Celuta S. Alviano, André L. S. Santos, Lucimar F. Kneipp

**Affiliations:** 1 Laboratório de Taxonomia, Bioquímica e Bioprospecção de Fungos (LTBBF), Instituto Oswaldo Cruz (IOC), Fundação Oswaldo Cruz (FIOCRUZ), Rio de Janeiro 21040-900, Brazil; 2 Laboratório de Estudos Avançados de Microrganismos Emergentes e Resistentes (LEAMER), Instituto de Microbiologia Paulo de Góes (IMPG), Universidade Federal do Rio de Janeiro (UFRJ), Rio de Janeiro 21941-901, Brazil; 3 Laboratório de Estrutura de Microrganismos, Instituto de Microbiologia Paulo de Góes (IMPG), Universidade Federal do Rio de Janeiro (UFRJ), Rio de Janeiro 21941-902, Brazil; 4 Rede Micologia RJ—Fundação de Amparo à Pesquisa do Estado do Rio de Janeiro (FAPERJ), Rio de Janeiro 20020-000, Brazil

**Keywords:** biofilm, antifungal resistance, dematiaceous fungi, efflux pumps, virulence

## Abstract

Patients with chromoblastomycosis (CBM) suffer chronic tissue lesions that are hard to treat. Considering that biofilm is the main growth lifestyle of several pathogens and it is involved with both virulence and resistance to antimicrobial drugs, we have investigated the ability of CBM fungi to produce this complex, organized and multicellular structure. *Fonsecaea pedrosoi* and *Phialophora verrucosa* conidial cells were able to adhere on a polystyrene abiotic substrate, differentiate into hyphae and produce a robust viable biomass containing extracellular matrix. Confocal laser scanning microscopy (CLSM) and scanning electron microscopy (SEM) showed the tridimensional architecture of the mature biofilms, revealing a dense network of interconnected hyphae, inner channels and amorphous extracellular polymeric material. Interestingly, the co-culture of each fungus with THP-1 macrophage cells, used as a biotic substrate, induced the formation of a mycelial trap covering and damaging the macrophages. In addition, the biofilm-forming cells of *F. pedrosoi* and *P. verrucosa* were more resistant to the conventional antifungal drugs than the planktonic-growing conidial cells. The efflux pump activities of *P. verrucosa* and *F. pedrosoi* biofilms were significantly higher than those measured in conidia. Taken together, the data pointed out the biofilm formation by CBM fungi and brought up a discussion of the relevance of studies about their antifungal resistance mechanisms.

## 1. Introduction

Chromoblastomycosis (CBM) is a neglected tropical disease characterized by the traumatic implantation of dematiaceous fungi in the human cutaneous and subcutaneous tissue [1]. CBM begins with wounds, especially on the upper and lower limbs, causing plaque, cicatricial, nodular and verrucous lesions, for instance. In addition to these extensive and long-lasting lesions, this disease can cause fibrotic and lymphatic damage that can generate chronic lymphedema with an elephantiasis aspect [1,2]. In severe cases, patients can also have squamous cell carcinoma. These clinical complications may lead to the amputation of the affected limb. Consequently, CBM is the cause of many morbidity cases worldwide, being considered an occupational disease that can permanently disable the infected individual [3,4]. Relevantly, CBM’s chronicity and recurrence combined with its fungal resistance to available therapies make it hard to treat [1,4]. This mycosis is ubiquitous, but it shows the highest prevalence rates in Africa, Asia and Latin America, particularly affecting people who are in contact with soil, wood and decaying materials, which are the natural habitats of CBM fungi [3,5]. The fungi belonging to the genus *Fonsecaea* and *Phialophora* are among the CBM etiological agents; however, *F. pedrosoi* is the most frequently diagnosed globally [4]. 

Although the pathogenesis mechanisms involved in CBM are still scarcely understood, several virulence attributes, such as melanin production, cell-surface and extracellular hydrolytic enzymes (e.g., peptidases and ecto-phosphatases), morphological transitions and adhesion to host cells have already been reported [1,6,7,8,9,10,11,12,13]. It is well-known that the fungal ability to adhere to the host tissue is one of the first and crucial steps for disease development [14,15]. In recent years, different groups have studied the capacity of filamentous fungi to form biofilms [16,17,18], an important virulence factor first well documented in yeasts of the *Candida* genus [19,20,21,22]. Biofilms are complex microbial communities defined by the presence of cells that may be attached to each other, to a substratum or embedded within an extracellular matrix containing polymeric substances produced by themselves [23]. The biofilm-forming ability gives the cells several advantages, such as increased virulence, immune system protection, tolerance to different environmental stressors, communication and metabolic cooperation, in addition to resistance to antimicrobials [17,23]. Thus, biofilm establishment is the predominant growth lifestyle of many pathogens, including filamentous fungi such as species belonging to the *Aspergillus*, *Scedosporium*, *Trichophyton, Fusarium, Trichosporon* and *Coccidioides* genera [24,25,26,27,28]. 

The production of microbial biofilm is a challenge for health professionals since these structures are formed on biotic surfaces, such as teeth, mucosal surface and chronic wounds, as well as on abiotic substrates including contact lenses, dental implants, different prosthetic valves and catheters, and other medical devices [29,30]. According to the U.S. National Institute of Health (NIH), almost 80% of chronic infections in humans are associated with biofilm formation [31]. Indeed, biofilm-related infections are resistant to drugs, demanding high medical care costs and prolonged stay in the hospital environment [23]. Different mechanisms of fungal resistance under biofilm conditions have been described in the literature [32,33,34]. A fundamental characteristic of biofilm resistance is the presence of extracellular matrix, mainly composed of polysaccharides, (glycol)proteins, (glycol)lipids, minerals, extracellular deoxyribonucleic acid (eDNA) and water, functioning as a protection for cells against the surrounding environment [32,35]. Under such conditions, the biofilm-forming cells secrete molecules known as quorum sensing inhibitors in order to establish the cell-to-cell communication that plays an important role in fungal resistance and pathogenicity [36]. Moreover, elevated efflux pump activity contributed to antifungal drug resistance. One of the most studied is the efflux pump-mediated resistance of *Candida* species, especially to azole drugs [37,38]. 

Some reports have shown that environmental dematiaceous fungi, including *Phialopora* spp., *Cladosporium* spp., *Alternaria* spp. and *Exophiala* spp., are able to produce biofilm-like structures on abiotic surfaces, especially in water distribution systems, contaminating domestic water taps [39,40,41,42]. The ability of dematiaceous pathogenic fungi to produce biofilm was described for *Exophiala dermatitidis*, the main agent of phaeohyphomycosis, which can also cause chronic diseases such as CBM [43,44,45]. In general, the results obtained for environmental and clinical isolates of *E. dermatitidis* varied widely; however, the latter showed a higher percentage of biofilm-forming strains [44,45]. Kirchhoff et al. [45] showed that invasive isolates of *E. dermatitidis* have a higher biofilm-forming ability than those frequently isolated from the respiratory tract of cystic fibrosis patients. Additionally, the ability of dematiaceous fungi to produce biofilm was also reported for *Cladosporium sphaerospermum*, which was isolated from patients with keratitis, and for clinical isolates of *Scedosporium* spp. and *Lomentospora prolificans* recovered from mycetoma [46,47,48]. Studies have shown that fungal biofilm has great clinical importance for chronic infections [49]. It is well recognized that chronic tissue lesions are colonized by polymicrobial communities, contributing to persistent inflammation, hindering the tissue regeneration process and significantly reducing patients’ quality of life as a consequence [50]. The environment of these chronic wounds favors polymicrobial biofilm formation and affects clinical treatment [50,51,52]. Remarkably, the occurrence of secondary bacterial infection is frequent in severe cases of CBM and makes its treatment even harder [1,53].

Based on all the above-mentioned premises, the present work initially investigated the ability of clinical isolates of *F. pedrosoi* and *P. verrucosa* to adhere to, differentiate and produce biofilm on a polystyrene surface. Moreover, the antifungal susceptibility and the activity of an ABC efflux pump in both conidial- and biofilm-forming cells were evaluated. Finally, the possible role of fungal efflux pumps on itraconazole resistance was tested by means of pharmacological inhibition.

## 2. Materials and Methods

### 2.1. The Fungi and Growth Conditions

Clinical isolates of *F. pedrosoi* (ATCC 46428, previously called 5VPL) and *P. verrucosa* (ATCC 28182) were provided by the collection of Reference Microorganisms in Sanitary Surveillance (CMRVS) of the National Institute for Quality Control in Health, Oswaldo Cruz Foundation (FIOCRUZ). Fungal isolates were maintained at 4 °C on Sabouraud dextrose agar (SDA) using mineral oil for preservation. For all the experiments, both fungi were grown at 26 °C for 6 days in a 100 mL of Czapek-Dox broth medium (BD-Difco, MD, USA) at pH 5.5 [54]. In order to obtain the conidial cells, the fungal cultures were filtered through gauze, centrifuged at 2400× *g* for 10 min and washed three times in 0.9% NaCl. Then, the number of conidial cells was determined using Neubauer chamber [54]. 

### 2.2. Adhesion and Germination Capability of Conidial Cells to Polystyrene Surface

Conidia (1 × 10^6^) were added to flat-bottom 24-well polystyrene plates (catalog number 92024 from TPP Techno Plastic Products AG, Trasadingen, Switzerland) containing 100 µL of Roswell Park Memorial Institute (RPMI) 1640 medium (Sigma-Aldrich, St. Louis, MO, USA) and incubated at 37 °C for 4 h (adhesion assay) and for 4, 8 and 12 h (germination assay). After washing three times with sterile phosphate-buffered saline (PBS, pH 7.2) to remove nonadherent fungi, the cells were observed through an inverted microscope (Nikon TS100-F, Tokyo, Japan) with a ×40 objective lens. The experiments were performed in triplicate, and five random fields per well of each system were considered to calculate the total number of fungi adhered on the polystyrene surface as well as the percentage of germinated cells arising from the adhesion process [47]. 

### 2.3. Biofilm Formation Detection on the Polystyrene Surface

Conidia (1 × 10^6^) were placed into flat-bottom 96-well polystyrene microtiter plates (catalog number 82.1581.001 from Sarstedt Inc., Nümbrecht, Germany) containing 100 μL of RPMI medium and incubated for 24, 48 and 72 h at 37 °C. For each plate, medium-only control wells were prepared. After incubations, nonadherent cells were removed, and the viability/metabolic activity, biomass and extracellular matrix were detected with colorimetric assays using a microtiter plate reader (SpectraMax M3Molecular Devices, Molecular Devices, LLC, San Jose, CA, USA). Briefly, the viability was assessed with 2,3-bis (2-methoxy-4-nitro-5-sulfophenyl)-5-[(phenylamino) carbonyl]-2H-tetrazolium hydroxide (XTT; Sigma-Aldrich) assay [24]. A solution containing 100 µL of XTT 0.04 mg and menadione 0.0005 mg was added to the plate wells and then incubated for 4 h in the dark at 37 °C. Then, the plates were subjected to centrifugation at 4000× *g* for 5 min, the supernatants transferred to other 96-well plates and the absorbance measured at 490 nm. Meanwhile, for biomass quantification, cells were fixed for 15 min with 100 μL of methanol, and after it was discarded, the wells were air-dried for 5 min. Then, they were stained with 100 μL of 0.3% crystal violet solution (Sigma-Aldrich) for 20 min. After that, cells were washed twice with PBS, incubated for 5 min with 100 μL of 30% acetic acid and then read at 590 nm [55]. For extracellular matrix detection, 100 μL of 0.1% safranin (Sigma-Aldrich) in PBS was added to the plate wells. Following 5 min at room temperature, the wells were washed and decolorized with 100 μL of 30% of acetic acid, and then the absorbance measured at 490 nm [22].

### 2.4. Confocal Laser Scanning Microscopy (CLSM) Analysis

Conidia (1 × 10^6^) were grown on polystyrene confocal plates (SPL Life Sciences Co., Pocheon, Korea) for 72 h at 37 °C and then stained for 1 h at 26 °C with Calcofluor white M2R (5 µg/mL, Sigma-Aldrich), FilmTracer SYPRO^®^ (200 µL, Molecular Probes, Invitrogen) and TOTO™-1 iodide (1 mM, ThermoFisher Scientific, Waltham, MA, USA). The wells were washed twice with PBS and covered with *n*-propyl gallate, and the fungal cells were observed using the confocal microscope (Leica TCS SP5 AOBS, Wetzlar, Germany). The three-dimensional (3-D) visualization of the biofilm architecture and thickness was assessed using Fiji software (ImageJ2, UW-Madison LOCI, Madison, WI, USA). *Z*-stacks were collected from five random fields for each biofilm [47,48]. 

### 2.5. Scanning Electron Microscopy (SEM) Assay

Conidia (1 × 10^6^) were added to sterile polystyrene coverslips (Agar Scientific, Stansted, UK) placed in 24-well polystyrene plates and incubated for 72 h at 37 °C. Then, cells were fixed in 2.5% glutaraldehyde in 0.1 M sodium cacodylate buffer (pH 7.2) at 4 °C overnight. Subsequently, the systems were washed with PBS, post-fixed in 2% osmium tetroxide for 2 h and dehydrated in a series of acetone solutions with concentrations varying from 25 to 100%. Next, cells were critical point-dried in CO_2_, coated with gold (20–30 nm), and observed using Zeiss EVO10 scanning electron microscope (Zeiss, Oberkochen, Germany) [47]. 

### 2.6. Biofilm Formation on THP-1 Cells

The human monocytic leukemia THP-1 cell line (ATCC TIB-202) was cultivated in 25-cm^2^ cell culture flasks containing RPMI 1640 medium and 10% heat-inactivated fetal bovine serum (Sigma-Aldrich) at 37 °C in a 5% CO_2_ atmosphere [13]. For the experiments, THP-1 cells (4 × 10^5^/mL) were added to 24-well polystyrene plates and grown in the same conditions as described above, except for the RPMI medium that was supplemented with 80 nM phorbol 12-myristate 13-acetate (PMA; Sigma-Aldrich), in order to induce their differentiation into macrophages. After 24 h, the THP-1 cells were washed three times with RPMI and incubated in fresh medium for an additional 24 h. Then, conidia (4 × 10^6^/mL) were placed to interact with macrophages for 48 h at 37 °C in an atmosphere of 5% CO_2_. After that, the systems were washed three times to remove nonadherent fungal cells and incubated at 26 °C, in the absence of light, with 5 μg/mL of the following fluorescent dyes: propidium iodide (Sigma-Aldrich) for 10 min and Calcofluor white (Sigma-Aldrich) for 60 min. System controls containing only THP-1 cells and THP-1 cells treated with paraformaldehyde at 4% for 30 min were also observed [47]. The damage to THP-1 cells provoked by fungal biofilm formation was monitored using a Zeiss LSM 710, AxioObserver confocal laser microscope with an oil-immersion EC Plan-Neofluar 40×/1.30 objective (Carl Zeiss Microscopy) and lasers 488 nm (propidium iodide) and 405 nm (calcofluor white). Images were assessed using ZEN 2.1 (black) software version number 14.0.25.201, Carl Zeiss Microscopy Company, Jena, Germany.

### 2.7. Antifungal Susceptibility Testing

This assay was performed according to the document M38-A2 of Clinical and Laboratory Standards Institute (CLSI) [56], with some modifications. Briefly, planktonic cells (1 × 10^4^) were placed into 96-well polystyrene microtiter plates containing RPMI 1640 buffered with 3-(*N*-morpholino) propanesulfonic acid (MOPS, Sigma-Aldrich) 0.165 mM, pH 7.0. After that, the antifungal agents amphotericin B, ketoconazole, itraconazole, posaconazole and terbinafine (Sigma-Aldrich), at concentrations varying from 0.048 to 100 µM, in 0.5% dimethylsulfoxide (DMSO, Sigma-Aldrich) were added. The plates were then incubated at 37 °C for 72 h. For the mature biofilm assays, 1 × 10^4^ conidia, initial inoculums, were added to 96-well plates with the same medium, and just after 72 h of incubation at 37 °C, it was supplemented with antifungal drugs at concentrations ranging from 0.39 to 800 µM. These plates were then incubated for additional 48 h. For susceptibility analysis, the minimum inhibitory concentrations (MIC_100_) of the planktonic cells and the biofilms (bMIC) were defined visually and confirmed by XTT-reduction assay as described above [24]. Systems containing RPMI medium and fungal cells supplemented or not with DMSO (final concentration of 0.5%) as well as having both RPMI and DMSO or only RPMI were used as controls.

### 2.8. Activity of ABC Efflux Pumps on Conidial- and Biofilm-Forming Cells 

Both conidial and 72 h-old-biofilm-forming cells (1 × 10^7^/mL, initial inoculums) were incubated with 10 µM rhodamine 6G (R6G, Sigma-Aldrich) for 1 h at 37 °C, as described by Silva et al. [57]. Then, the supernatants were discarded, and the systems were washed with PBS and incubated for additional 1 h at 37 °C in PBS containing 2% glucose. The supernatants were then collected, and fluorescence was measured on a fluorimeter (excitation, 529 nm; emission, 553 nm). R6G non-stained fungal cells were used as a control. In addition, *F. pedrosoi* and *P. verrucosa* were treated with MIC (both 0.19 µM) determined for planktonic cells and with the highest concentration (800 µM) tested before for biofilm-forming cells. The efflux pump inhibitor phenylalanine-arginine beta-naphthylamide (PAβN, Sigma-Aldrich) was used to evaluate the possible role of efflux pumps in antifungal resistance. The bMIC for itraconazole in the presence of PAβN (64 µg/mL) was determined after 48 h of incubation according to CLSI [56], with modifications. 

### 2.9. Statistical Analysis

All experiments were performed in three independent experimental sets. All graphics and statistical analyses were performed with GraphPad Prism 5.0 software (GraphPad Software, Inc., La Jolla, CA, USA). Data were expressed as mean ± standard deviation. 

## 3. Results and Discussion

### 3.1. Adhesion and Germination of Fungi on Polystyrene Substrate

It is well-known that at an early stage of biofilm formation, fungal cells adhere to non-living (abiotic) or living (biotic) substrates through the interactions of electrostatic forces with their cell wall-associated adhesion molecules [58,59]. Several aspects such as contact surface nature, environmental factors and fungal morphology can influence the formation and structure of biofilm [23]. Different types of inert substrates have been used for testing biofilm formation [60]. In this study, we selected polystyrene since it is the classical substrate used to demonstrate this multicellular structure produced by microbial cells, including fungi in vitro [24,25,47], which permits the comparison of biofilm formation capability among different strains, species and genera. Moreover, the analytical methods for studying biofilm using polystyrene as substrate are easy, low-cost and reproducible [60].

**Figure 1:**
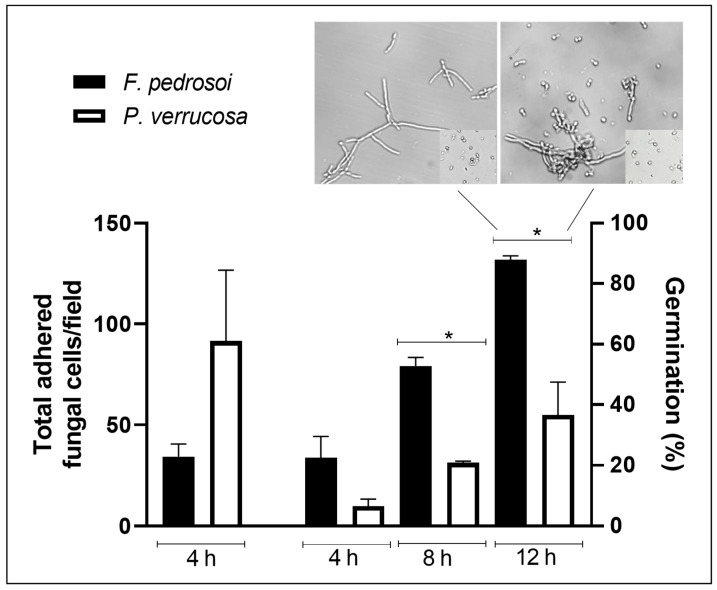
Fungal adhesion and germination on a polystyrene substrate. Fungal cells (1 × 10^6^) were incubated at 37 °C in a 24-well polystyrene plate containing RPMI medium. The wells were washed, and then the total adhered cell numbers per field after 4 h of incubation and the percentage of conidial germination at 4, 8 and 12 h were determined using the inverted microscope. Asterisks represent statistical significance (*p* < 0.05). Images show fungal cells adhered to polystyrene after 12 h of germination. Inset: conidial cells before the adhesion process.

Therefore, we first investigated the ability of *F. pedrosoi* and *P. verrucosa* conidial cells to adhere on an abiotic surface that is chemically composed of polystyrene. Our results showed that after 4 h of fungi-polystyrene contact, the total adhered cell numbers per field for *F. pedrosoi* and *P. verrucosa* were 34.2 ± 4.4 and 86.3 ± 25.0, respectively (Figure 1). In addition to adhesion, the capability to germinate is an important characteristic of biofilm establishment. Studies have highlighted that filamentation is directly related to biofilm development and fungal pathogenicity [61,62]. Both fungi germinated in contact with polystyrene in a typically time-dependent manner (Figure 1). Although *P. verrucosa* showed higher adhesion to polystyrene, its germination process was less efficient than that observed in *F. pedrosoi* at all time points studied (Figure 1). For instance, *F. pedrosoi* presented a higher percentage of conidial germination (87.9 ± 0.9%) after 12 h of interaction with polystyrene compared with *P. verrucosa* (36.6 ± 7.7%) (Figure 1). A similar lag phase was reported for *A. fumigatus* biofilm formation, which just germinated on a polystyrene plate after approximately 10 h of incubation [24,63]. After abiotic surface contact, the ability of conidial cells for adhesion, germination and differentiation into mycelia was also reported for other clinically relevant filamentous fungi, including *Scedosporium apiospermum, S. aurantiacum* and *L. prolificans* [47,64]. 

### 3.2. Measurement of Classical Biofilm Parameters

**Figure 2:**
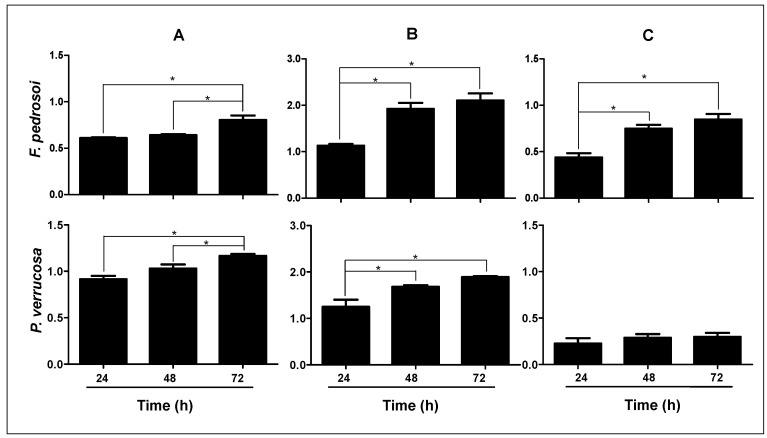
Biofilm formation by *F. pedrosoi* and *P. verrucosa* on a polystyrene surface. Fungal cells (1 × 10^6^) were incubated for 24, 48 and 72 h at 37 °C in 96-well polystyrene plates containing RPMI medium. After incubations, the following parameters were assessed: (**A**) cell viability using XTT reduction assay (490 nm), (**B**) biomass after the incorporation of crystal violet in methanol-fixed biofilm (590 nm), and (**C**) extracellular matrix after the incorporation of safranin in non-fixed biofilm (590 nm). The data were expressed as means ± SDs. Asterisks represent statistical significance (*p* < 0.05).

Structural characteristics of microbial biofilms, such as biomass, viability and extracellular matrix, were assessed in *F. pedrosoi* and *P. verrucosa* using classical colorimetric assays [50]. Firstly, the cellular viability determined on XTT assay (Figure 2A) revealed that biofilm-forming *F. pedrosoi* and *P. verrucosa* cells were capable of efficiently reducing the tetrazolium salt. Both fungi reached the highest metabolic activity after 72 h of contact with the polystyrene surface. These viable fungal cells were able to produce a robust biomass, as judged by the incorporation of crystal violet in the methanol-fixed biofilm (Figure 2B), as well as extracellular matrix through the absorption of safranin in non-fixed biofilm (Figure 2C). The biomass of *F. pedrosoi* and *P. verrucosa* as well as the amount of extracellular matrix for *F. pedrosoi* reached their maximum at 48 h of incubation under the employed experimental conditions, while the *P. verrucosa* extracellular matrix remained constant for all the analyzed periods. Studies have shown that the quantity of extracellular matrix as well as biomass can be strain- and/or species-specific [45,65]. Kirchhoff et al. [45] investigated the ability of 58 clinical strains of *E. dermatitidis* to form biofilm. Those authors reported that the invasive isolates recovered from non-cystic fibrosis patients exhibited significantly more biomass than did the isolates recovered from cystic fibrosis patients. Interestingly, the clinical isolates of *C. albicans* recovered from bloodstream and vaginal mucosa produce distinct biomass that determine if they are high or low biofilm producers, affecting their antifungal susceptibility [66]. Mello et al. [47] studied biofilm formed by *Scedosporium* spp. and *L. prolificans* and showed that the biomass and extracellular matrix produced by *S. aurantiacum* and *S. minutisporum* were significantly higher than those detected for *S. apiospermum* and *L. prolificans*, directly impacting the antifungal resistance pattern.

### 3.3. Fungal Biofilm Structural Distribution and 3-D Organization

**Figure 3:**
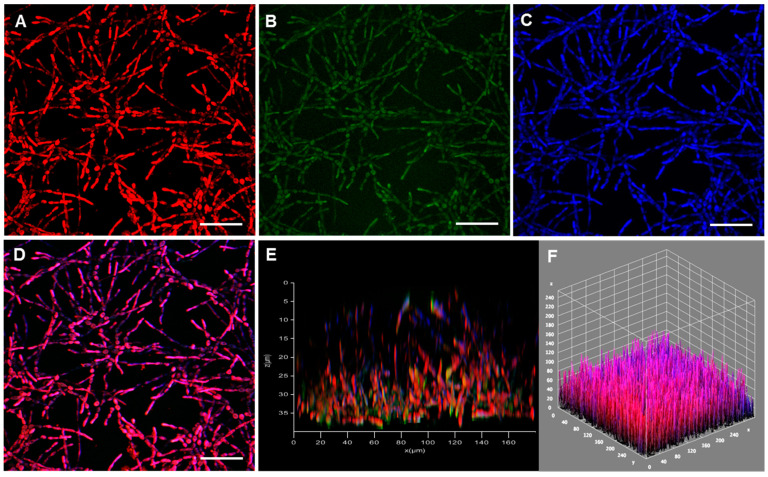
Confocal microscopy of the biofilm formed by *F. pedrosoi* cells on polystyrene. Conidia (1 × 10^6^) were incubated at 37 °C in polystyrene confocal plates containing RPMI medium. After 48 h, the fungal cells were stained with (**A**) FilmTracer SYPRO^®^, (**B**) TOTO^TM^, (**C**) calcofluor white M2R and (**D**) the staining combined. (**E**,**F**) 3-D reconstructions of the fungal biofilm. Bars, 20 μm.

**Figure 4:**
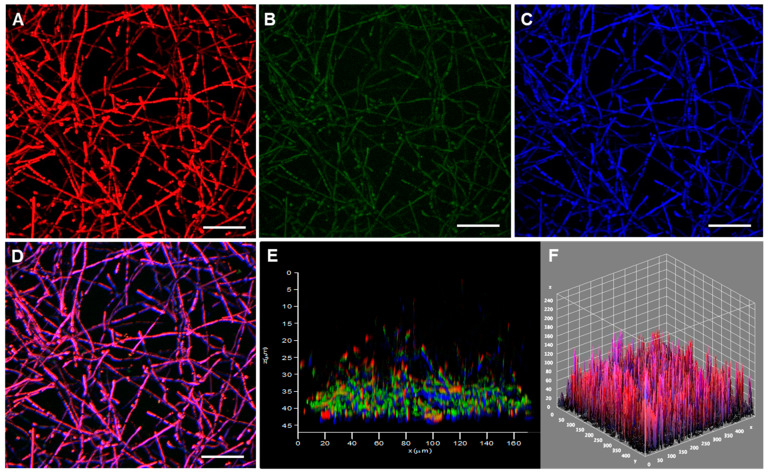
Confocal microscopy of the biofilm formed by *P. verrucosa* cells on polystyrene. Conidia (1 × 10^6^) were incubated at 37 °C in polystyrene confocal plates containing RPMI medium. After 48 h, the fungal cells were stained with (**A**) FilmTracer SYPRO^®^, (**B**) TOTO^TM^, (**C**) calcofluor white M2R and (**D**) the staining combined. (**E**,**F**) 3-D reconstructions of the fungal biofilm. Bars, 20 μm.

Several imaging methodologies have been applied in biofilm studies, including CLSM, a nondestructive technique used in combination with different fluorescent dyes [67] that was also used here to analyze the 3D biofilm architecture of CBM fungi (Figure 3 and Figure 4). In this context, the extracellular matrix was observed using the FilmTracer SYPRO^®^, which stains glycoproteins, highlighting this crucial structural component of the biofilms formed by both *F. pedrosoi* (Figure 3A) and *P. verrucosa* (Figure 4A). The results herein corroborated the data demonstrated on colorimetric assays and were similar to those obtained from CLSM for the qualitative observation of other fungal biofilms [48,68]. Several groups have described the relevance of extracellular matrix in the biofilm of different fungi. This structure acts as a protective barrier; holds the cells together; and contributes to biofilm architecture, integrity and mechanical stability [35,69] Subsequently, the presence of eDNA, an important component of biofilm extracellular matrix in CBM fungi, was checked after staining with TOTO^TM^ 1-iodide. The results revealed the presence of eDNA spread in the extracellular matrix trap that forms the biofilm structure of *F. pedrosoi* (Figure 3B) and *P. verrucosa* (Figure 4B). Studies showed that the eDNA released by *C. albicans* clinical isolates is variable and can be involved with differential biofilm formation [70]. In fact, the authors demonstrated that the greater eDNA levels in *C. albicans* and *A. fumigatus* isolates were able to form more robust biofilms than the other isolates that formed thin biofilms [70,71]. Overall, the functions proposed to be attributable to the presence of eDNA in the biofilm architecture include maintaining the biofilm structural integrity and being directly involved with antifungal resistance, since some antifungals covalently bind to the eDNA [71,72]. 

Moreover, our data showed that calcofluor white M2R, which binds to chitin in the fungal cell walls, exhibited the outlines of *F. pedrosoi* and *P. verrucosa* hyphae, the main structure that composes their biofilm biomass (Figure 3C and Figure 4C). Studies with biofilm-producing *Candida* species revealed differences in cellular morphologies and in the extracellular matrix production [69]. Overall, mature *C. albicans* biofilm has a complex structure formed by blastophores, yeasts and hyphae embedded in a dense extracellular matrix [73]. In contrast, the biofilm produced by *C. glabrata* is especially formed of yeast cells [74], similar to the *C. parapsilosis* biofilm, which is composed of a small amount of extracellular matrix [20]. These differences in the biofilm structural composition determine its shape and architecture [75]. Our data revealed that the thicknesses of the 3-D biofilm architecture formed by *F. pedrosoi* and *P. verrucosa* cells were 35 and 45 μm, respectively (Figure 3E,F and Figure 4E,F). Kirchhoff et al. [45] demonstrated with CLSM that *E. dermatitidis* biofilm thickness was strain-dependent. Those authors showed that the biofilm formed by invasive isolates had more hyphae and were thicker than those formed by isolated recovered from cystic fibrosis patients. The thickness of the mature biofilm of *F. pedrosoi* and *P. verrucosa* was lower than that measured for *P. brasiliensis* (100 µm) and *C. neoformans* (76 μm), but it was greater than that observed for the invasive isolate of *E. dermatitidis* (~10 µm). While *P. verrucosa* had a higher thickness rate than the clinical isolates of *Candida haemulonii* complexes, which ranged from 21.6 to 39.1 μm [22,76,77,78]. It is known that biofilm thickness can change according to some physicochemical conditions including substrate composition, nutritional conditions and environmental influences. For instance, Martinez et al. [79] showed that in vivo *C. albicans* mature biofilms can be thicker than those obtained in in vitro (laboratorial) conditions.

### 3.4. Ultrastructure of Biofilm-Growing Cells

**Figure 5:**
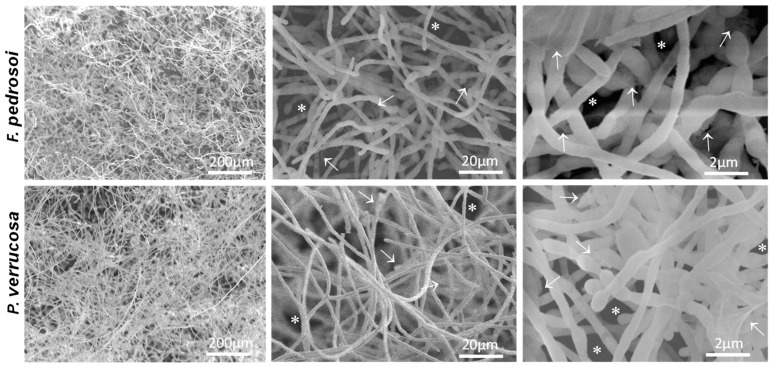
Low-magnification SEM images of fungal biofilm on a polystyrene surface. Fungal cells (1 × 10^6^) were added to polystyrene cover slips and incubated for 72 h at 37 °C. Then, the cells were processed for SEM, as detailed in Material and Methods. Representative images of *F. pedrosoi* and *P. verrucosa* showed biofilm features as dense masses of mycelia containing inner water channels (asterisks) and extracellular matrix (white arrows).

**Figure 6:**
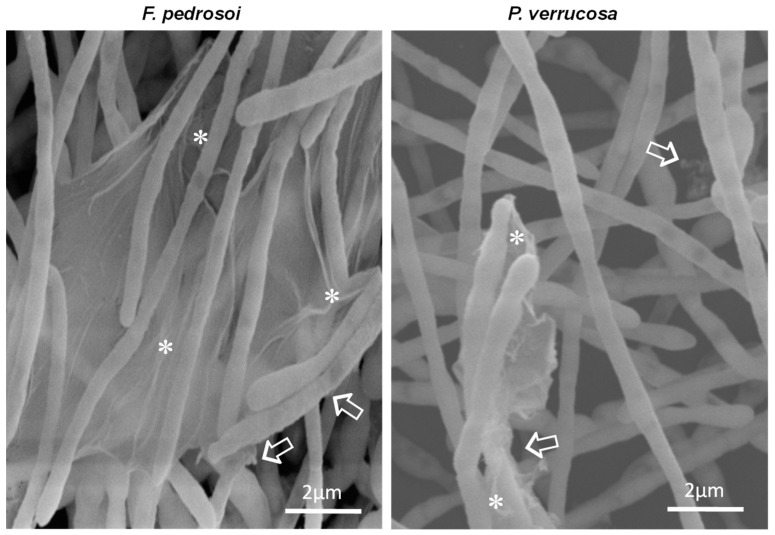
High-magnification SEM images of fungal biofilm on a polystyrene surface. The representative images show the extracellular matrix around the hyphal cells (white arrows) and also acting as glue that holds the hyphae attached (asterisks).

Another microscopy technique for fungal biofilm investigation is the SEM, which has been used to detail its morphology and architecture [80]. Here, SEM images of biofilms formed by *F. pedrosoi* and *P. verrucosa* revealed a compacted and dense mycelial trap characterized by the presence of entangled and interconnected cells (Figure 5). Inner water channels and extracellular matrix interlaced with the hyphae of both fungi were also observed (Figure 5). Studies have shown that these channels favor nutrient and water passage as well as cell dispersion [16]. The extracellular matrix detection corroborated the data found in CLSM analysis and showed that *F. pedrosoi* and *P. verrucosa* have common biofilm structural characteristics, following the pattern of other already described fungal pathogens including *P. brasiliensis, A. fumigatus, Scedosporium* spp., *L. prolificans*, *T. mentagrophytes* and *T. rubrum* [25,47,76,81]. The high-magnification SEM images (Figure 6) showed that the extracellular matrix morphology was in general distinct and presented a structure like a veil for *F. pedrosoi* and a fluffy aspect for *P. verrucosa.* Regarding the biofilm extracellular matrix, SEM revealed that it was arranged around the *F. pedrosoi* and *P. verrucosa* hyphae or bound to them (Figure 6). The matrix can promote adherence, nutrient capture and protection against surrounding stresses, including UV radiation and desiccation as well as antimicrobial agents’ action [82]. 

### 3.5. Biofilm Formation on Animal Cells 

**Figure 7:**
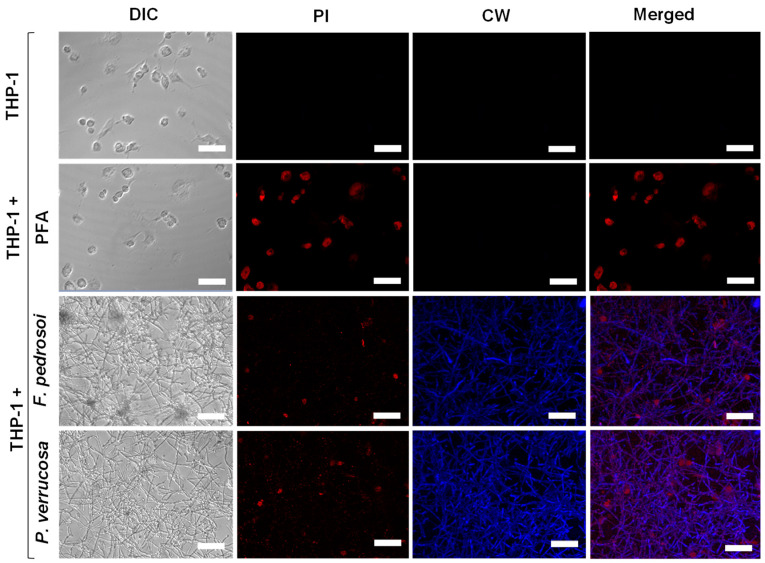
Confocal microscopy of fungal biofilm formation on THP-1 cells. The human monocytic leukemia cell line (4 × 10^5^/mL) was added in 24-well cell culture plates containing RPMI medium supplemented with 80 nM PMA to differentiate macrophage cells, as detailed in Material and Methods. Representative images of THP-1 cells non-treated (control of viable cells), treated with paraformaldehyde (PFA, control of non-viable cells) or incubated for 48 h with (4 × 10^6^/mL) of *F. pedrosoi* and *P. verrucosa*. After co-culturing, the systems were washed with RPMI and incubated with propidium iodide (PI) and calcofluor white (CW). THP-1 cells damage by fungal biofilm formation was monitored using confocal differential interference contrast (DIC) and fluorescence microscopy. Bars, 50 μm.

The ability of fungi to adhere and form biofilm on a biotic substrate was also investigated using macrophages derived from human monocytic lineage THP-1 cells. We demonstrated that *F. pedrosoi* and *P. verrucosa* conidia were able to adhere to THP-1 and differentiate into mycelia, yielding a multicellular structure resembling a biofilm, as detected with calcofluor white, which stains chitin and highlights the hyphal cells forming the biofilm biomass (Figure 7). Our results are in agreement with those of Katragkou et al. [83], who also used THP-1 cells as an in vitro biofilm model and showed that *C. albicans* adhered to the macrophage monolayer and formed a biofilm. In fact, during invasive infections, many biofilm-producing pathogens can proliferate on the surface of epithelial, endothelial and immune host cells [84,85]. Thus, authors concluded that *Candida* biofilms were more resistant against the action of monocytes, changing their cytokine profile as well as reducing the migratory capacity of macrophages [42,83]. In addition, macrophages are not efficient against mature *Candida* biofilm and might even boost biofilm formation [86]. The ability to produce biofilm on human and cystic fibrosis bronchial epithelial cells was reported for *A. fumigatus* [84]. Mello et al. [47] demonstrated that the conidia of *Scedosporium* spp. and *L. prolificans* adhered and produced a typical biofilm structure with a dense mycelial mass covering the adenocarcinome human alveolar basal epithelial cells (A549). After this interaction, *L. prolificans* in particular was able to destroy the epithelial cells. Similarly, the interaction of *F. pedrosoi* and *P. verrucosa* with THP-1 cells revealed that these CBM fungi affected the viability of macrophages in comparison with the uninfected macrophage cells, as detected by propidium iodide staining (Figure 7).

### 3.6. Susceptibility of Planktonic and Biofilm Cells to Antifungal Agents

Biofilm is considered a resistant structure against antimicrobial drugs. In the present study, we assessed the resistant properties of biofilm formed by *F. pedrosoi* and *P. verrucosa* to distinct antifungal classes, including azoles (ketoconazole, itraconazole and posaconazole), polyene (amphotericin B) and allylamine (terbinafine) (Table 1). The results highlighted that the MIC values for all tested antifungals were higher in biofilm-forming cells than in the planktonic counterparts in both *F. pedrosoi* or *P. verrucosa*. These data corroborate previously published results that showed greater biofilm antifungal resistance of yeasts like *Candida* spp. [87] and filamentous fungi such as *E. dermatitidis*, *A. fumigatus*, *Fusarium solani* and *Scedosporium* spp. than of their planktonic counterparts [45,47,88,89]. In general, most biofilm-forming cells have been up to 1000-fold more resistant to antifungal agents than planktonic cells [32]. We observed this profile (bMIC > 1000× compared with MIC) when *F. pedrosoi* and *P. verrucosa* were treated with itraconazole, in which bMIC was higher than the maximum concentration (800 µM) tested. Itraconazole was 1000 times less efficient against the mature biofilm compared with the planktonic cells of *A. fumigatus* [24]. *E. dermatitidis* biofilm resistance against azoles, like itraconazole and voriconazole, was also previously reported [43,44,45,90]. Similar results were observed for the biofilm of *F. pedrosoi* after treatment with either terbinafine or posaconazole (bMIC > 800 µM) (Table 1). Additionally, for *P. verrucosa*, we observed bMIC > 800 µM to terbinafine, while posaconazole had a bMIC (400 µM) 40,000-fold higher than MIC. In parallel, bMIC to ketoconazole for *F. pedrosoi* (800 µM) and *P. verrucosa* (400 µM) increased 2051- and 8-fold, respectively, compared with their planktonic MIC values. Likewise, azoles’ ineffectiveness against the biofilms of *Candida* spp., *Cryptococcus neoformans* and *A. fumigatus* was demonstrated by different research groups [77,88,91]. In addition, our results showed a lesser effect of amphotericin B on the bMIC of both fungi, increasing 8-fold for *P. verrucosa* and 32-fold for *F. pedrosoi* (Table 1).

It is important to mention that our findings corroborate the relevance of searching for new drugs to treat fungal infections such as CBM and to standardize drug susceptibility assays against biofilm instead of conventional assays that used only planktonic cells. Recently, we evaluated the effects of metal-based drugs on *P. verrucosa* under biofilm conditions [92]. The data showed that 1,10-phenanthroline-5,6-dione (phendione) and its metal-based complexes, [Ag(phendione)_2_]ClO_4_ and [Cu(phendione)_3_](ClO_4_)_2_.4H_2_O, were able to disturb the mature biofilm formed by *P. verrucosa*, presenting bMIC with concentrations (96, 128 and 20 µM, respectively) lower than itraconazole (>800 µM). Likewise, metal-based nanoparticles showed *C. albicans* antibiofilm activity. For instance, both silver and copper nanoparticles were able to inhibit *C. albicans* biofilm formation [93,94]. In fact, studies have proposed strategies to treat biofilm-associated infections, especially those caused by *C. albicans*, such as new biomaterials with anti-adhesive properties and small molecule-based chemical approaches [95]. In addition, the drug repositioning represents one of the strategies against fungal biofilms [96].

### 3.7. Activity of Efflux Pumps on Conidia and Biofilm-Forming Cells

**Figure 8:**
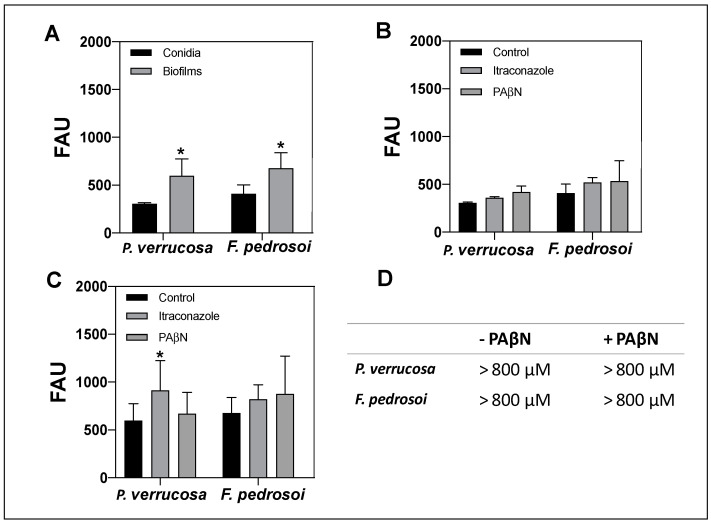
Activity of efflux pumps on conidia and biofilm-forming cells of *F. pedrosoi* and *P. verrucosa*. (**A**) Conidial cells and 72 h-old-biofilm (1 × 10^7^/mL, initial inoculums) were first incubated with rhodamine 6G (R6G, 10 µM) and then with glucose 2% in order to evaluate the glucose-induced efflux of R6G. (**B**) Conidia and (**C**) mature biofilm pretreated for 1 h with MICs of itraconazole or with phenylalanine-arginine beta-naphthylamide (PAβN, 64 µg/mL), before the addition of R6G and glucose. (**D**) Susceptibility assay of biofilm-forming cells treated for 48 h with itraconazole in the absence or the presence of PAβN determined with CLSI [56]. Data were expressed as fluorescence arbitrary unit (FAU) after a fluorimeter reading (excitation, 529 nm; emission, 553 nm). Symbol (*) represents *p* values ≤ 0.05 compared with control.

In order to understand the key factors involved with the inefficiency of antifungal drugs against biofilms, we studied the efflux pump activity since it is also associated with the resistance of biofilm-forming cells, as described previously for *Candida* spp. and *A. fumigatus* [57,97]. In fungal cells, biofilm resistance is multifactorial and can also be attributed to drug sequestration and its limited diffusion by extracellular matrix, which functions as a molecular trap, as well as to the reduction of drug access to their target by efflux pump action [33,35]. The efflux pumps that mediate the antifungal resistance are frequently related to the transmembrane transporter, especially the ATP-binding cassette (ABC) transporter family [37,38]. In this set of experiments, the ABC efflux pump activity was assessed using the glucose-induced efflux of R6G method (Figure 8). This fluorescent dye is first taken up by fungal cells, and then, after adding glucose, the cells that have efflux pump activity throw R6G out, which is detected in the supernatant. Thus, the levels of intracellular R6G accumulation indirectly indicate the pump efflux activity. Our data showed that the efflux pump activity of *P. verrucosa* and *F. pedrosoi* biofilms was significantly higher than that of their conidia (Figure 8A). Itraconazole was chosen since it is the most commonly used antifungal drug in CBM therapy. The pretreatment with itraconazole and PAβN, an efflux pump inhibitor, did not affect the efflux pump activity of conidia (Figure 8B). Rangel et al. [98] showed the presence of an ABC transporter in the plasma membrane of *F. pedrosoi* using Western blot analysis with anti-Pdr5p antibody. The authors also demonstrated, by reverse transcription polymerase chain reaction (RT-PCR), that *F. pedrosoi* grown for ~15 days with sublethal concentrations (1 and 4 µg/mL) of itraconazole had the expression of its ABC transport gene stimulated. In contrast, our data revealed that under the conditions tested, itraconazole only induced a significant increase in the efflux pump activity of *P. verrucosa* biofilm (Figure 8C). Several studies showed that antifungal drugs like azoles may be substrates for efflux pumps, inducing their overexpression and culminating in fungal resistance [17,37]. Indeed, clinical isolates of *A. fumigatus*, *C. albicans* and *C. neoformans*, which are azole-resistant, exhibit transcriptional activation of efflux pump encoding genes that can reduce intracellular drugs accumulation to lethal levels and lead their extrusion and tolerance [37].

Furthermore, our data revealed that PAβN did not change the bMIC values of *P. verrucosa* and *F. pedrosoi* (Figure 8D). Thus, this efflux pump inhibitor was not able to reverse the itraconazole resistance phenotype of biofilm-growing cells. Additional experiments need to be conducted to clarify the resistance mechanisms involved with this antifungal drug. Overall, the data of the present study indicated that neither the inhibitor PAβN nor itraconazole may have reached the fungal cells in a concentration capable of affecting the ABC efflux pump. In addition to ABC, another drug transporter belongs to this superfamily of proteins, called major facilitator superfamily (MFS) may be responsible for *F. pedrosoi* and *P. verrucosa* resistance to itraconazole like reported for *C. albicans* [61,99]. We also cannot rule out that the efflux pumps do not take part in the itraconazole resistance presented by biofilm-producing CBM fungi. Ramage et al. [61] showed that the major mechanism of *C. albicans* azole resistance is mediated by ABC and MFS transporters. However, for this, yeast efflux pumps are not important in amphotericin B’s and echinocandins’ resistance [91]. In fact, studies have highlighted that antifungal biofilm resistance requires distinct molecular mechanisms from those already established for planktonic cells [61,91].

## 4. Conclusions

In the present work, both *F. pedrosoi* and *P. verrucosa* were able to produce networks of filamentous forms with entangled and interconnected cells on a polystyrene surface, as found in the classic microbial biofilm formed by other pathogenic filamentous fungi. These special features, including the ability to adhere, produce extracellular matrix and increase the antifungal drug resistance, corroborated the potential biofilm formation by these CBM fungi. The ABC efflux pump activity of *P. verrucosa* and *F. pedrosoi* biofilms was significantly higher than that of their conidia. Future studies are necessary to clarify the resistance mechanism of the biofilm formed by these fungi and show its impact on CBM chronicity, which may contribute to achieving more effective treatment of this debilitating infection.

## Figures and Tables

**Table 1:** The susceptibility of planktonic- and biofilm-forming cells of *F. pedrosoi* and *P. verrucosa* to antifungal agents.

	*F. pedrosoi* MIC (µM/mg/L)			*P. verrucosa* MIC (µM/mg/L)		
Antifungal Agents	Planktonic	Biofilm	bMIC/MIC	Planktonic	Biofilm	bMIC/MIC
Amphotericin B	6.25/5.78	200/185	**↑** 32×	3.12/2.88	25/23	**↑** 8×
Ketoconazole	0.39/0.21	800/425	**↑** 2051×	50/27	400/213	**↑** 8×
Itraconazole	0.19/0.13	>800/>560	**↑** >4200×	0.19/0.13	>800/>560	**↑** >4200×
Posaconazole	0.04/0.03	>800/>600	**↑** >20.000×	0.01/0.007	400/280	**↑** 40.000×
Terbinafine	0.78/0.23	>800/>230	**↑** >1000×	0.04/0.01	>800/>230	**↑** >20.000×

Planktonic cells (1 × 10^4^) and mature biofilm (initial inoculum of 1 × 10^4^) were treated with different antifungal agents. The minimum inhibitory concentration (MIC) was defined as 100% growth inhibition using visual inspection as recommended by CLSI [56] and confirmed with XTT colorimetric assay [24]. The calculation considered the minimum drug concentration not able to promote the XTT reduction, representing cells metabolically inactivate and/or nonviable. The MIC was expressed in both micromolars (µM) and milligrams per liter (mg/L). (↑) Represents the order of magnitude, in which biofilm MIC (bMIC) was higher than the MIC of planktonic cells. Biofilm-forming cells of *F. pedrosoi* and *P. verrucosa* showed significantly higher MICs (*p* < 0.05) than planktonic cells for all tested antifungal agents.

## Data Availability

Not applicable.
